# Skull Density Ratio as Arm‐Allocation Parameter for a Controlled Focused Ultrasound Trial in Parkinson's Disease

**DOI:** 10.1002/mdc3.14040

**Published:** 2024-05-13

**Authors:** José Angel Pineda‐Pardo, Raul Martínez‐Fernández, Elena Natera‐Villalba, Agustina Ruiz‐Yanzi, Rafael Rodríguez‐Rojas, Marta Del Alamo, Tamara Jiménez‐Castellanos, Michele Matarazzo, Carmen Gasca‐Salas, Olivier Rascol, José A. Obeso

**Affiliations:** ^1^ HM CINAC MADRID (Centro Integral de Neurociencias Abarca Campal). Hospital Universitario HM Puerta del Sur, HM Hospitales Madrid Spain; ^2^ CIBERNED, Instituto de Salud Carlos III Madrid Spain; ^3^ Instituto de Investigación Sanitaria HM Hospitales Spain; ^4^ PhD Medicine Program Universidad Autonoma de Madrid Madrid Spain; ^5^ Facultad HM de Ciencias de la Salud de la Universidad Camilo José Cela Madrid Spain; ^6^ Department of Preventive Medicine Public Health and Microbiology, Universidad Autónoma de Madrid Madrid Spain; ^7^ Universidad San Pablo‐CEU Madrid Spain; ^8^ Clinical Investigation Center CIC 1436, NS‐Park/F‐CRIN Network and NeuroToul COEN Center; Inserm University of Toulouse 3 and CHU of Toulouse F‐31000 Toulouse France

**Keywords:** focused ultrasound, skull density ratio, clinical trials, Parkinson's disease, subthalamotomy

## Abstract

**Background:**

MR‐guided focused ultrasound (FUS) thermoablation is an established therapy for movement disorders. FUS candidates must meet a predefined threshold of skull density ratio (SDR), a parameter that accounts for the efficiency in reaching ablative temperatures. Randomized sham‐controlled trials to provide definitive therapeutic evidence employ pure randomization of subjects into active treatment or control arms. The latter design has several general limitations.

**Objective:**

To demonstrate that SDR values are not associated with clinically and demographically relevant variables in patients with Parkinson's disease (PD). This in turn would allow using SDR as an arm‐allocation parameter, separating patients who will receive active FUS treatment and best medical management treatment (BMT).

**Methods:**

We studied a cohort of 215 PD patients who were candidates for FUS subthalamotomy to determine if the SDR was correlated with demographic or clinical variables that could introduce bias for group allocation in a controlled trial.

**Results:**

SDR was unassociated with age, gender, and clinical motor features nor with levodopa daily dose in our cohort of PD patients. A negative association with age was found for the female subgroup.

**Conclusions:**

Our results show that in a PD population considered for FUS subthalamotomy treatment, the SDR may be a valid group‐allocation parameter. This could be considered as the basis for a controlled study comparing FUS subthalamotomy vs BMT.

## Introduction

MR‐guided focused ultrasound (FUS) thermoablation is an effective therapy for movement disorders such as essential tremor (ET) and Parkinson's disease (PD). Furthermore, newer questions and possible newer indications may arise as FUS methodology continues to develop and expand.

Typically, providing high‐level scientific evidence of efficacy for a new therapy requires a randomized controlled trial. However, using a sham‐controlled arm in trials of interventional therapies such as FUS gives rise to some relevant concerns. Thus, FUS sham procedures are not hazard‐free and some relevant complications can occur.^1^ Moreover, the high symptomatic effect of FUS therapeutic thermoablation compromises blinding for both patients and evaluators which becomes impossible to maintain for long follow‐ups.

To overcome these limitations, we propose using the skull density ratio (SDR) as the group allocation method. Accordingly, there would be two arms, active FUS treatment vs. best medical treatment (BMT) only. The SDR is a widely used parameter to predict the ability of ultrasound energy to pass through an intact skull and the most validated measure to predict the likelihood of reaching therapeutic temperatures on target (ie, thermal efficiency).^2^, ^3^, ^4^ Typically, regardless of the condition, eligibility for FUS treatment in a given patient requires the SDR to be above a pre‐established threshold. SDR ranges from 0 to 1, where lower scores indicate higher attenuation of ultrasound energy and, consequently greater unsuitability for FUS treatment. Hence, an SDR cut‐off could be contemplated as a tool to separate patients who fulfill clinical criteria for an intention to treat analysis of a controlled study.

Towards this aim, it is necessary to prove that SDR values are not associated with any relevant clinical variable. In other words, that SDR values do not signal and separate out different underlying demographic or disease characteristics.

In order to use SDR cut‐offs as an allocation strategy we have ascertained whether or not SDR values show any relationship with main clinical or demographic features in a large cohort of PD patients who were considered potential candidates for FUS treatment of the subthalamic nucleus (FUS‐STN). We have centered the analysis on STN ablation (ie, subthalamotomy) because it is the primary target for functional neurosurgical treatments of PD worldwide and we have carried out several studies with this approach.^5^, ^6^, ^7^

## Methods

### Patients

A sample of 215 consecutive PD patients who attended our movement disorders clinics and were considered candidates for FUS‐STN in the period between December 2015 and April 2022 were included in this study. Patients were assessed at HM CINAC in Hospital HM Puerta del Sur, Madrid, Spain. Indication for ultrasound ablation was based on the presence of asymmetrical parkinsonism which was not adequately controlled in the more affected body side despite the use of optimized anti‐parkinsonian medication.^8^ All patients underwent brain CT scan for SDR calculation as part of the standard FUS treatment screening process.

### Data Acquisition and SDR Estimation

The CT images were obtained using a Toshiba Acquilion Prime scanner with a selected number of slices to cover the entire head, extending from at least 1 cm above the head and beyond the skull base. The images were then reconstructed with a 512 × 512 matrix, 1 mm slice thickness, and an “FC30” convolution kernel, with contrast enhancement for bone tissues. The SDR values were estimated using the ExAblate Neuro (Insightec Ltd., Haifa, Israel) platform, after uploading the CT images. The anterior commissure (AC) and posterior commissure (PC) were identified and marked on the CT image, and an additional midline coordinate was marked in a coronal view to define the mid‐sagittal plane. A target corresponding to the STN location was then placed 12 mm lateral to the mid‐sagittal plane, 3 mm posterior to the mid‐commissural point, and 4 mm inferior to the AC‐PC line.^5^ A virtual simulated transducer array was positioned so that the natural focus coincided with the selected target and tilted around the right–left axis to avoid covering the frontal sinus. Finally, the SDR score, which quantifies the average ratio of CT intensities between the diploe and cortical bones across ultrasound beam trajectories^2^, ^4^ was obtained from the ExAblate software (version 7.0) and recorded for all subjects.

### Statistics

Two‐sample Student's *t*‐test was used for group statistics. Pearson linear correlations were estimated to determine the relationship between SDR values and clinical scores. Partial correlations were also assessed to control for time‐related variables that could affect clinical severity or levodopa equivalent daily dose (such as age, age at disease onset, and disease duration). We further explored clinical independence of SDR as a group allocation parameter using an iterative procedure. The patient cohort was split into two groups based on SDR values, taking a range of SDR cut‐offs that spanned the interquartile range of the entire cohort. For each cut‐off, two patient groups arise, namely below or above the SDR threshold, which we will call *Low* and *High* SDR groups for simplicity. To evaluate differences in clinical scores between these groups, a two‐sample Student's *t*‐test was conducted. Furthermore, we focused our analysis on a specific SDR cut‐off value of 0.42, which we considered appropriate to assign patients to FUS or best medical treatment (BMT) groups in a hypothetical FUS‐STN trial. The FDA‐approved and clinically recommended SDR cut‐off value in patient selection for treatment with FUS thalamotomy is 0.40. According to our database of PD patients clinically eligible for FUS‐STN, an SDR value of 0.39 falls within the 25th percentile of the sample distribution, while a value of 0.42 falls within the 33rd percentile. Therefore, if the former score is used as the assignment cut‐off, it would result in a distribution of patients with a ratio of 3:1 (active treatment: control), whereas the latter would yield a ratio of 2:1. To ensure that all patients included in the treatment arm are indeed suitable candidates for FUS (ie, their SDR value is above 0.4), we selected a 2:1 distribution (commonly used in FUS trials^6^, ^9^:) corresponding to an SDR cut‐off value of 0.42. In this case, a two‐sample Student's *t*‐test was also used to test for differences between below‐threshold (BMT) and above‐threshold (FUS) groups.

A *P‐value* of <0.05 was utilized to determine statistical significance in all conducted tests. Additionally, Bayesian analyses were employed for all group comparisons and correlation tests, with Bayes factors being reported using JASP software.^10^ Bayes factors, denoted as BF_10_, represent the degree of evidence supporting the alternative hypothesis (H_1_) compared to the null hypothesis (H_0_), being H_0_ the absence of difference between group means, or the absence of correlation between variables. Bayes factors are interpreted as follows: BF ≤3 = weak evidence; BF between 3 and 10 = moderate evidence; BF above 10 = strong evidence. Similarly, the degree of evidence in favor of the null hypothesis can be assessed using the reciprocal of the Bayes factor (BF_01_ = 1/BF_10_), which is interpreted in the same way.

## Results

### SDR and Clinical Features of PD

**Figure 1:**
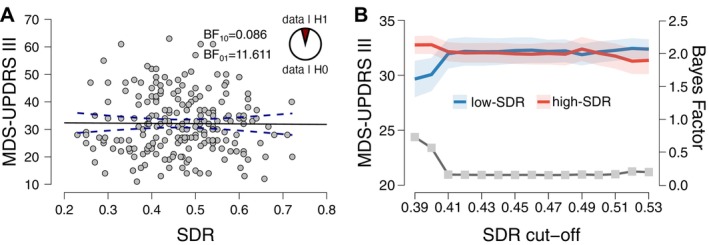
SDR and parkinsonism are independent. A scatterplot (**A**) shows MDS‐UPDRS part III scores vs. SDR with Bayes Factor indicating strong evidence for the null‐hypothesis. MDS‐UPDRS scores (**B**) stratified by SDR cut‐offs (*P < 0.05).

Among this sample of 215 patients, the mean (SD) SDR was 0.47 (0.10) with a full‐range spanning [0.23–0.72] and interquartile range [0.39–0.54]. A significant negative association between age and SDR was found in females r = −0.37 (*P* = 0.0014; BF_10_ = 21.763). Pearson linear correlations between motor impairment in the off‐medication state according to the Movement Disorders Society‐Unified Parkinson's Disease rating scale (MDS‐UPDRS‐III) and SDR values did not show a significant association in the full cohort (r = 0.009; *P* > 0.05; BF_10_ = 0.086, Fig. 1A), thus providing strong evidence supporting the null‐hypothesis, ie, absence of a correlation (BF_01_ = 11.611). In addition, SDR did not correlate with LEDD in a non‐selected sub‐group (*n* = 49) of our patients (r = −0.045; *P* > 0.05; BF_10_ = 0.190). Including age and disease duration as controlling factors did not alter the results. Additionally, we tested the between‐group difference (ie, below‐SDR cut‐off value vs above‐SDR value) in the MDS‐UPDRS III scores along the range of SDR spanning the interquartile range [0.39–0.54]. No significant difference in motor severity between groups was found for any comparison (*P*‐value >0.05), and Bayes Factors were consistently below 1 in all tests (Fig. 1B).

When specifically considering 0.42 as the cut‐off value for arm allocation, the two groups obtained (*Low*‐ and *High* SDR groups) did not show differences in either demographic (age, gender) or clinical variables (MDS‐UPDRS III, age of onset, or disease duration) (*P* > 0.05, BF_10_ < 1) (see Table 1).

**TABLE 1:** Demographic characteristics of PD patients grouped by the 0.42 SDR cut‐off of value.

Variables	(SDR > =0.42) (*N* = 142)	(SDR <0.42) (*N* = 73)	*P*‐value	BF_10_
Age	59.5 (10.4) [35–82]	61.5 (9.2) [41–84]	0.180	0.364
Gender	47/95 F/M	25/48 F/M	0.866	0.230
MDS‐UPDRS‐III Off‐medication	32.2 (10.5) [12–63]	32.0 (11.5) [11–61]	0.900	0.158
Age at onset	54.6 (10.2) [31.0–80.0]	56.6 (9.1) [35.8–76.9]	0.161	0.394
Disease duration [years]	4.9 (3.7) [0.2–20.6]	5.0 (3.9) [0.2–19.5]	0.924	0.157

Age, gender, clinical impairment with MDS‐UPDRS part III, age at disease onset and disease duration are reported for both cohorts. Data are shown as mean and standard deviation [MEAN (SD)] and range [MIN MAX]. *P*‐values are represented in the third column after Student's *t*‐test and chi‐squared test for gender. Bayes Factors (BF_10_) for the same comparisons are included in the fourth column.

## Discussion

This study shows that the SDR value is not correlated with any of the main demographics and clinical characteristics in a large cohort of PD patients who were considered potential candidates for FUS‐STN treatment. Interestingly, in our population, an SDR value of 0.42 emerges as adequate threshold parameter for a 2:1 ratio of allocation (FUS vs BMT). When considering a 0.42 value threshold, both arms were clinically identical (Table 1). By demonstrating the independence between SDR scores and PD motor severity, among other characteristics, we validate SDR segregation as a possible method for arm allocation in FUS controlled trials in PD.

We believe that the approach proposed here has several practical and conceptual advantages. The first and foremost advantage is avoidance of any undesirable adverse event potentially associated with the FUS sham procedure. These include the discomfort and pain caused by frame placement and pin‐site complications, nausea, and high blood pressure while in the MR machine.^6^, ^11^, ^12^ Although these complications are transient by nature (they are related to the procedure and the device), they can be significant.^1^ Secondly, in a future FUS controlled trial, patients would be screened out and enrolled based upon their compliance with the clinical inclusion criteria and willingness to participate. Subsequently, the SDR would be ascertained, and patients will be assigned to their corresponding trial arm. The majority of those with a *Low* SDR who would not be, in any case, suitable candidates for FUS‐STN, would benefit from continuous specialized medical attention, treatment optimization, and careful evaluations over a prolonged period at the BMT arm. Oppositely, all patients with a suitable SDR would receive active treatment after enrollment, which would not likely be the case if a standard randomized‐controlled design was applied. The latter is more meaningful when considering long‐term follow‐up studies. In a randomized trial, a number of subjects who are good candidates for FUS treatment would be falling into the sham arm. With disease progression, a percentage of them who are unable to be properly controlled only with medication would probably drop out from the study. Admittedly, patients with SDR between 0.3 and 0.4 may nowadays be treated,^13^ although at the cost of low thermal efficiency.^3^ Our experience^5^, ^6^, ^7^ with FUS‐STN suggests that an SDR below 0.4 is difficult to achieve adequate treatment and, in our practice, we rarely admit patients with lower SDRs for FUS‐STN treatment.

We should acknowledge several limitations associated with our approach. It is well‐known that SDR distribution has ethnic variability and that its value changes depending on the intended brain target. Thus, SDR outcome independence and cut‐off values for arm assignment cannot be universally defined. Therefore, in order to apply this method in a given project, the dissociation of each condition‐specific clinical characteristic from skull properties should be assessed beforehand. Moreover, while variability across CT systems is compensated during SDR estimation, other sort of bias might be introduced across centers (eg, observer induced bias) that might be relevant if considering a multi‐center study. This should be compensated by centralizing SDR estimations in a single reference center. Another limitation is that our analysis has concentrated in showing the independence of the SDR with motor clinical severity evaluated through the MDS‐UPDRS part III and LEDD. Hence, it is possible that other MDS‐UPDRS items and clinical aspects assessed with different scales could unravel some relationships. However, the motor MDS‐UPDRS is the most widely used primary outcome in PD trials and, accordingly, will be the primary efficacy outcome in most upcoming eventual studies. Also, a negative significant correlation was found in women between age and SDR that was not present in men. Aged women have a higher prevalence of osteoporosis,^14^ which by causing trabecular bone demineralization, and consequent attenuation of CT intensities^15^ might be mainly responsible for this finding. As patient age for clinical trial inclusion is usually restricted to a specific range, this aspect would be minimized. Last but not least, placebo effects in the FUS arm will not be ruled out because patients in the control arm will not be receiving any sham procedure. However, as reported in the literature,^6^, ^11^ most patients included in FUS randomized trials are aware of their assignment. Therefore, this is not a methodological limitation of the assignation method here described but of any randomized trial applying a very clinically impactful therapy. To minimize this bias in a clinical trial, the possibility of including a blinded rater to assess the main outcomes can be contemplated as a convenient additional feature.

Finally, it is worth mentioning that although we have circumscribed our proposal of using the SDR as an allocation parameter for a FUS‐STN trial, this approach can be extended to other PD targets or other disorders. Notably, open label FUS studies and isolated case reports have demonstrated preliminary efficacy and safety in the treatment of various sorts of tremor, focal dystonia, neuropathic pain, and psychiatric conditions such as obsessive‐compulsive disorder and major depression.^16^, ^17^, ^18^ Our approach for using SDR as a control arm allocation parameter could significantly minimize the undesirable effects of randomization and sham FUS procedures to further study those conditions.

In sum, we propose here to use the SDR as an assignment parameter for FUS controlled clinical trials. Our data support that this is a valid allocation method in PD that avoid difficulties and nuisances of typical sham‐procedure randomized trials. Admittedly, our design does not allow pure randomization and the implicit limitations must be considered; these apply particularly to studies aiming to generate data valid for regulatory and qualification agencies. Our findings should be expanded to other FUS targets and different populations worldwide in order to judge the variability and potential limitations of this approach.

## Author Roles

(1) Research project: A. Conception, B. Organization, C. Execution; (2) Statistical Analysis: A. Design, B. Execution, C. Review and Critique; (3) Manuscript Preparation: A. Writing of the first draft, B. Review and Critique.

J.A.P.P.: 1A, 1B, 1C, 2A, 2B, 3A

R.M.F.: 1A, 1B, 1C, 2A, 3A

E.N.V.: 1C, 3B

A.R.Y.: 1C, 3B

R.R.R.: 1C, 2C, 3B

M.d.A.: 1C, 3B

T.J.C.: 1C, 3B

M.M.: 1C, 2C, 3B

C.G.S.: 1C, 3B.

O.R.: 2A, 2C, 3B

J.A.O.: 1A, 1B, 1C, 2A, 2C, 3A

## Disclosures

**Ethical Compliance Statement:** The protocol adhered to all relevant regulations for human study participants, in accordance with the Declaration of Helsinki. Informed consent was obtained from all patients before CT scan imaging. We confirm that we have read the Journal's position on issues involved in ethical publication and affirm that this work is consistent with those guidelines.

**Funding Sources and Conflict of Interest:** This study was supported by Fundación de Investigación HM Hospitales, Fundación MAPFRE, by the Ministerio de Ciencia e Innovación (PID2021‐127800OA‐I00 – JAPP) and by Instituto de Salud Carlos III (PI21/00925 – RMF).

**Financial Disclosures for the Previous 12 Months:** JAPP has received speaker honoraria from Zambon. RMF has received speaker honoraria from Insightec, Bial, Palex and Zambon, and reimbursement of travel expenses to attend scientific conferences from Insightec. ENV has received honoraria for lectures from Zambon. ARY has received speaker honoraria from Zambon. RRR has received speaker honoraria from Zambon. MdA has received speaker honoraria from Insightec and Boston Scientific and reimbursement of travel expenses to attend scientific conferences from Boston Scientific and Medtronic. TJC has received speaker honoraria from Zambon. MM has received speaker honoraria from Teva Pharmaceutical Industries and Novartis and reimbursement of travel expenses to attend scientific conferences from Lundbeck and Cerevel Therapeutics. CGS has received speaker honoraria from Exeltis, Esteve and Fundación ACE, and a grant from Asociacion Madrileña de Neurologia, funded by Bial. OR has acted as scientific advisor/consultant for AbbVie, Adamas, Acorda, Addex, Aguettant, Alkahest, AlzProtect, Apopharma, Astrazeneca, Bial, Biogen, Britannia, Buckwang, Cerevel, Clevexel, Irlab, Eli‐Lilly, Lundbeck, Neuroderm, ONO Pharma, Orion Pharma, Osmotica, Oxford Biomedica, Pfizer, Prexton Therapeutics, Sanofi, Servier, Sunovion, Théranexus, Takeda, Teva, UCB, Watermark Research, XenoPort, XO, Zambon, and has received scientific grants from Agence Nationale de la Recherche (ANR), CHU de Toulouse, France‐Parkinson, INSERM‐DHOS Recherche Clinique Translationnelle, MJFox Foundation, Programme Hospitalier de Recherche Clinique, European Commission (FP7, H2020, Horizon Europe). JAO has received honoraria for lecturing and reimbursement of travel expenses to attend scientific meetings by Insightec.
